# Supplementary material for: Rapid host strain improvement by in vivo rearrangement of a synthetic yeast chromosome
Source: Nat Commun. 2018 May 22;9:1932. doi: 10.1038/s41467-018-03143-w (PMC5964169; doi:10.1038/s41467-018-03143-w)
Supplement: Supplementary file 1 — Supplementary Information [file 41467_2018_3143_MOESM1_ESM.pdf]

Rapid host strain improvement by *in vivo* rearrangement of a synthetic yeast chromosome

Blount *et al*.

Supplementary Information

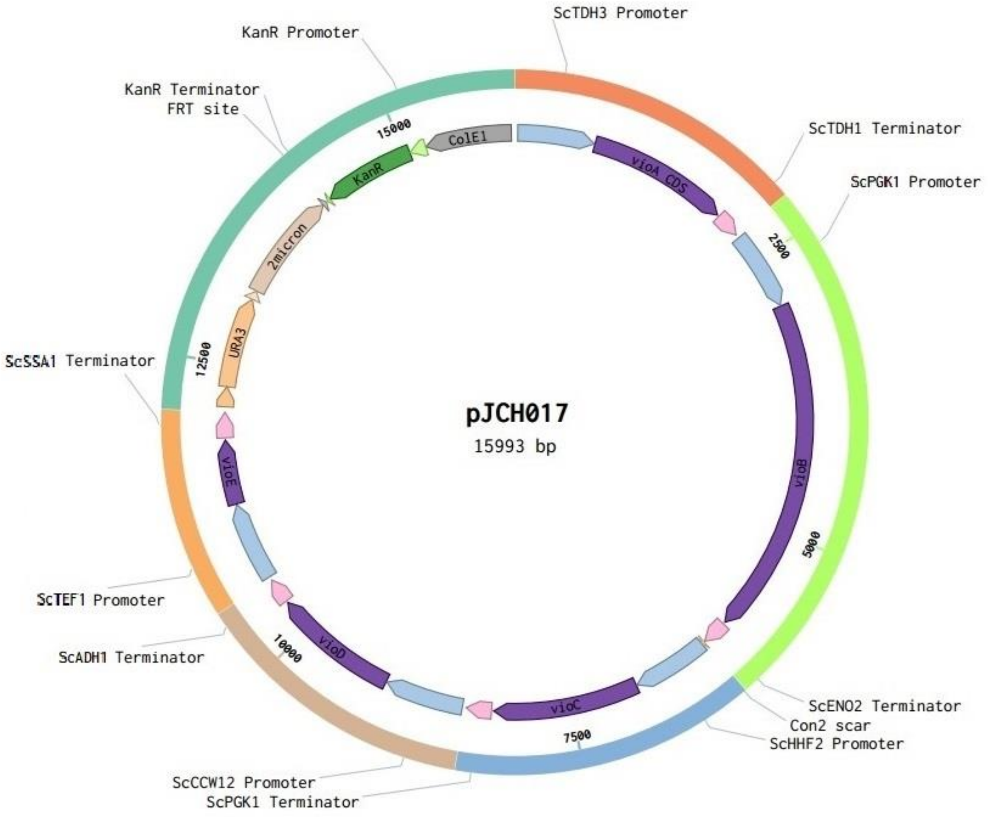

Supplementary Figure 1: Map of plasmid pJCH017. The plasmid map was generated using Benchling (benchling.com). Inner boxes represent plasmid feature and outer boxes represent sub-assembly pieces that were used to construct the plasmid.

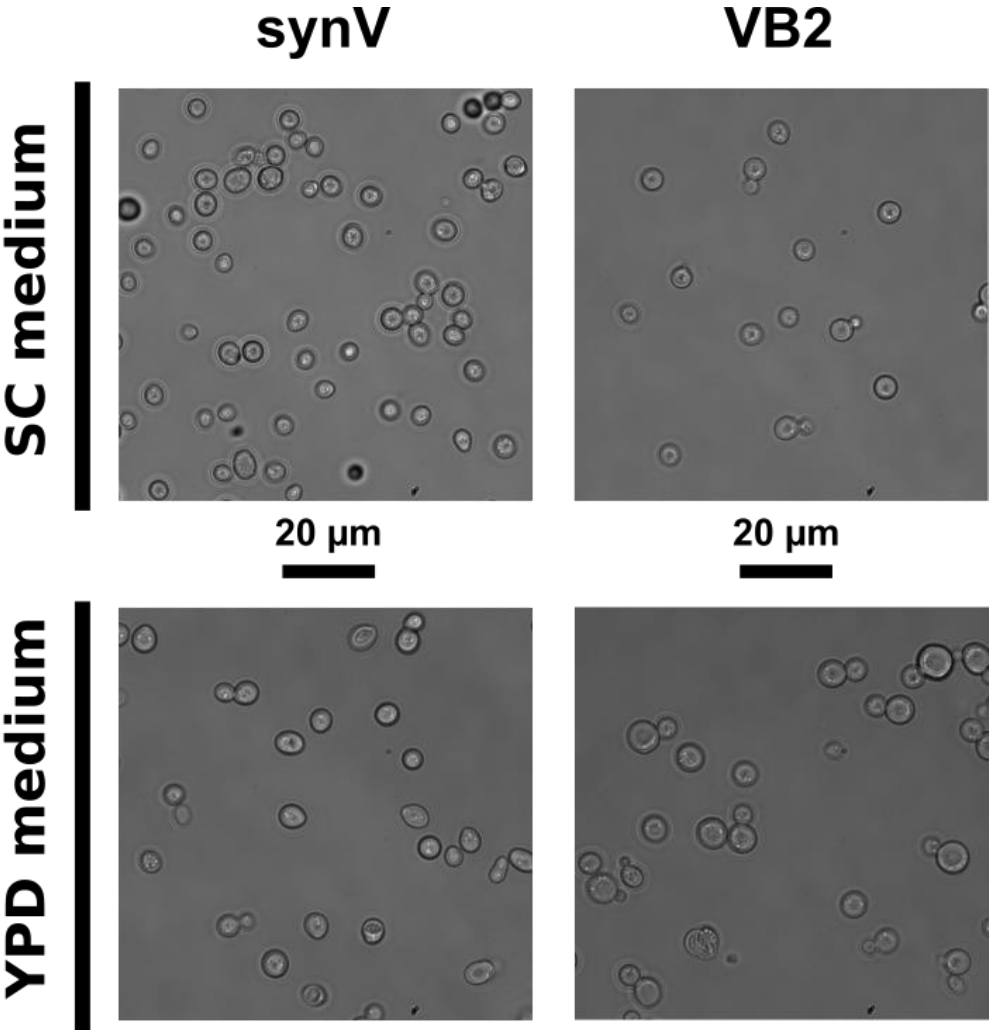

Supplementary Figure 2: Microscopy of strain VB2. Representative microscopy images of synV and VB2 cultures grown to stationary phase and visualised at 90x magnification.

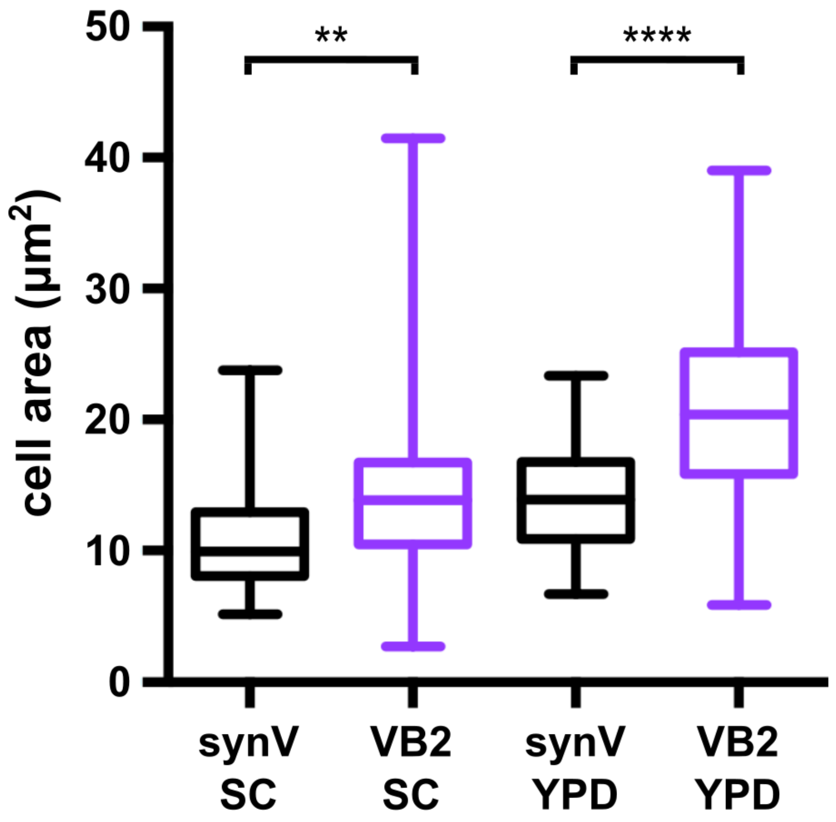

Supplementary Figure 3: VB2 cell area analysis. VB2 and synV cells visualised as in Figure S2 were assessed for cell area. SC denotes that the cells were grown in synthetic complete medium and YPD denotes that the cells were grown in YPD medium. Top and bottom box edges show the 75th and 25th percentiles with the line between showing the mean average. Bars show the minimum and maximum range of the data points, n≥24 cells. Asterisks denote two-tail p value as determined by two-sample t-test, with * denoting p≤0.05, ** denoting p≤0.01, *** denoting p≤0.001 and **** denoting p≤0.0001.

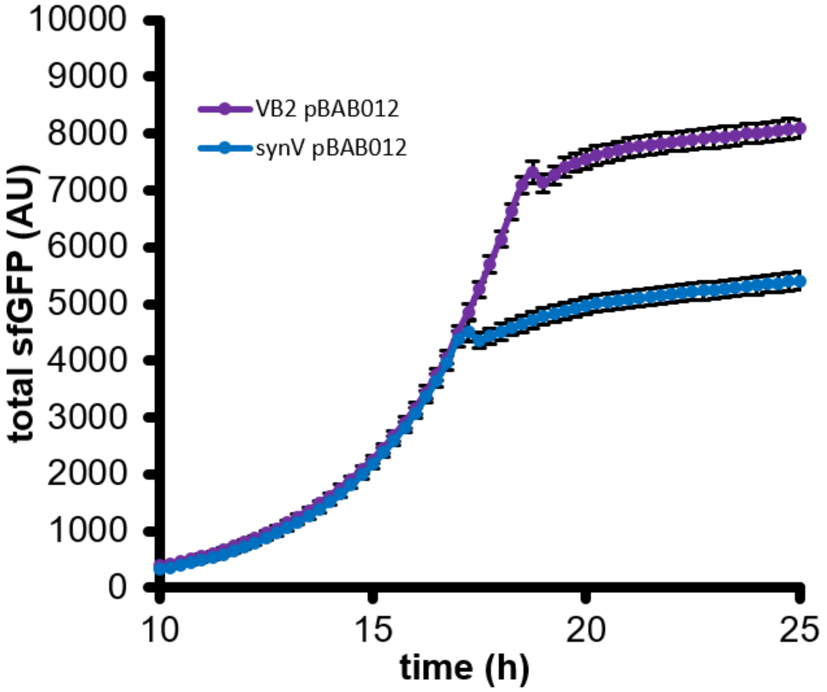

Supplementary Figure 4: Total sfGFP of VB2-pJCH017 cultures. synV-pBAB012 (sfGFP, 2μ vector) and VB2-pBAB012 were grown in 96-well format at 30 °C. Data points represent the mean fluorescence vale at 520 nm corrected for medium and host strain fluorescence. Error bars denote standard deviation, n≥7.

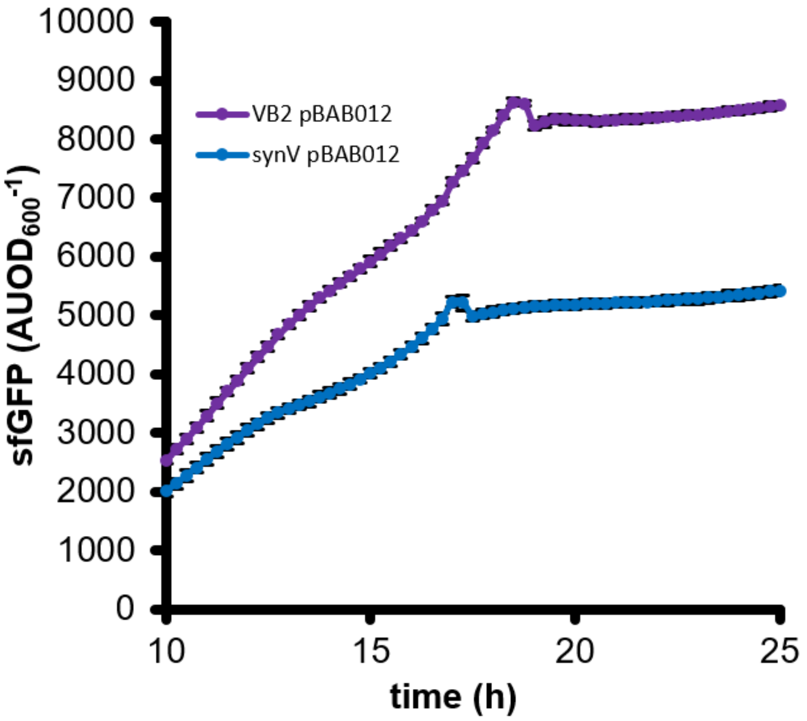

Supplementary Figure 5: sfGFP production of VB2 normalised to OD_{600}. synV - pBAB012 (sfGFP, 2µ vector) and VB2-pBAB012 were grown in 96-well format at 30 °C. Data points represent the mean fluorescence vale at 520 nm divided by the OD_{600} of the culture at that time point. All OD_{600} values were corrected for medium OD_{600} and all fluorescence values were corrected for medium and host strain fluorescence. Error bars denote standard deviation, n≥7.

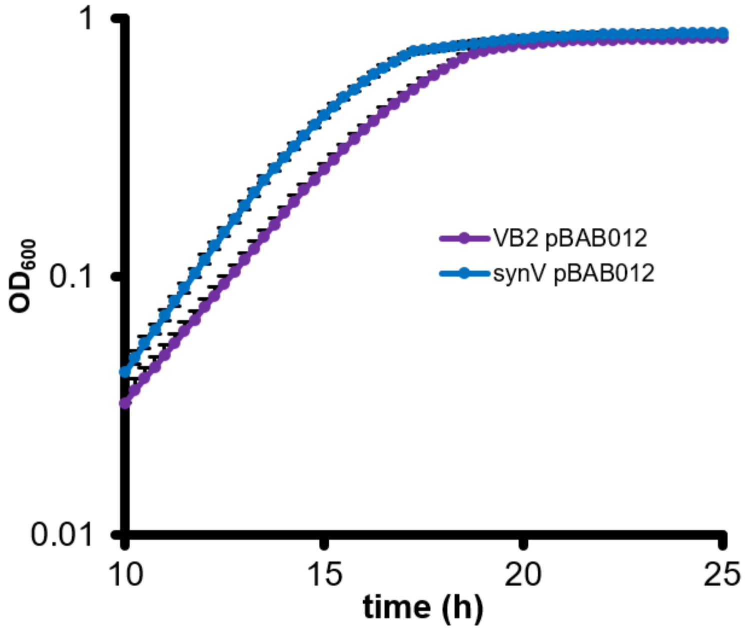

Supplementary Figure 6: Growth of synV and VB2 with pBAB012. synV-pBAB012 and XD4-pBAB012 cultures were grown in SDO URA- medium in 96-well format. Data points represent the mean OD_{600} vale at for medium OD_{600}. Error bars show standard deviation, n≥7.

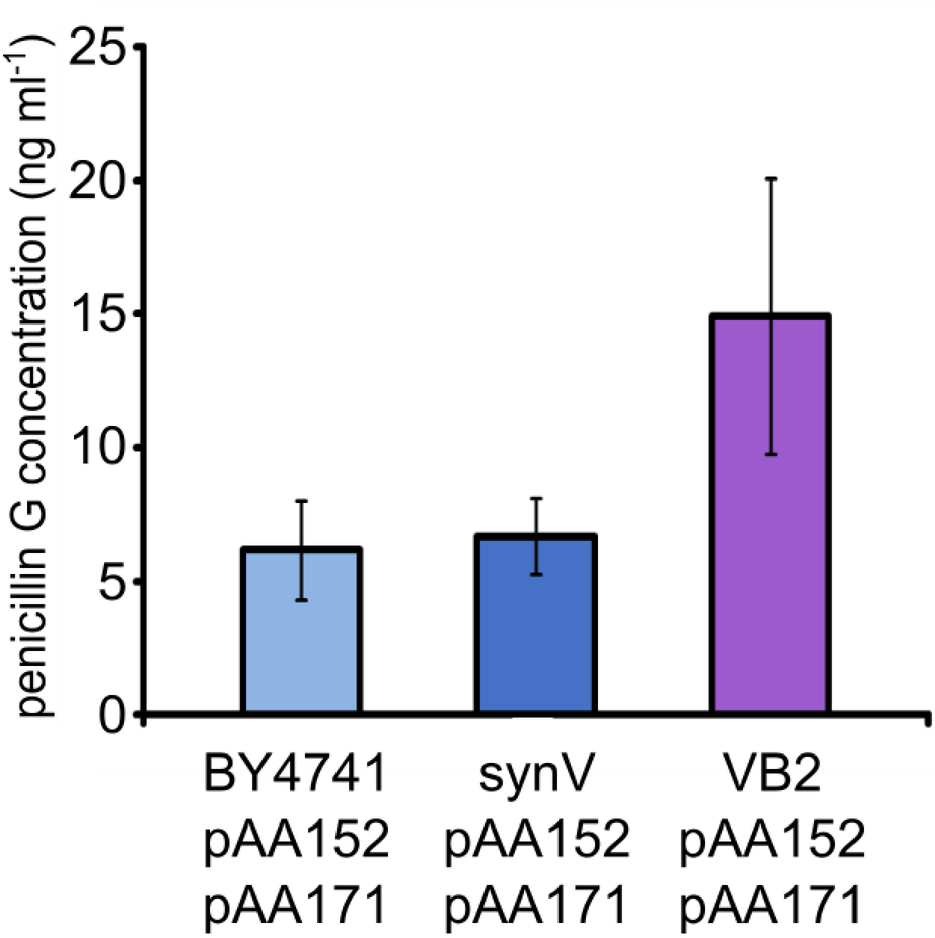

Supplementary Figure 7: VB2 penicillin production. Concentrations of penicillin G secreted by BY4741-pAA152/171, synV-pAA152/171 and VB2-pAA152/171 strains as determined by LC-MS. The data is the same as shown in Figure 2g except for without normalisation to OD_{600}, n=3. All values plotted are mean averages and error bars represent 1 standard deviation from the mean.

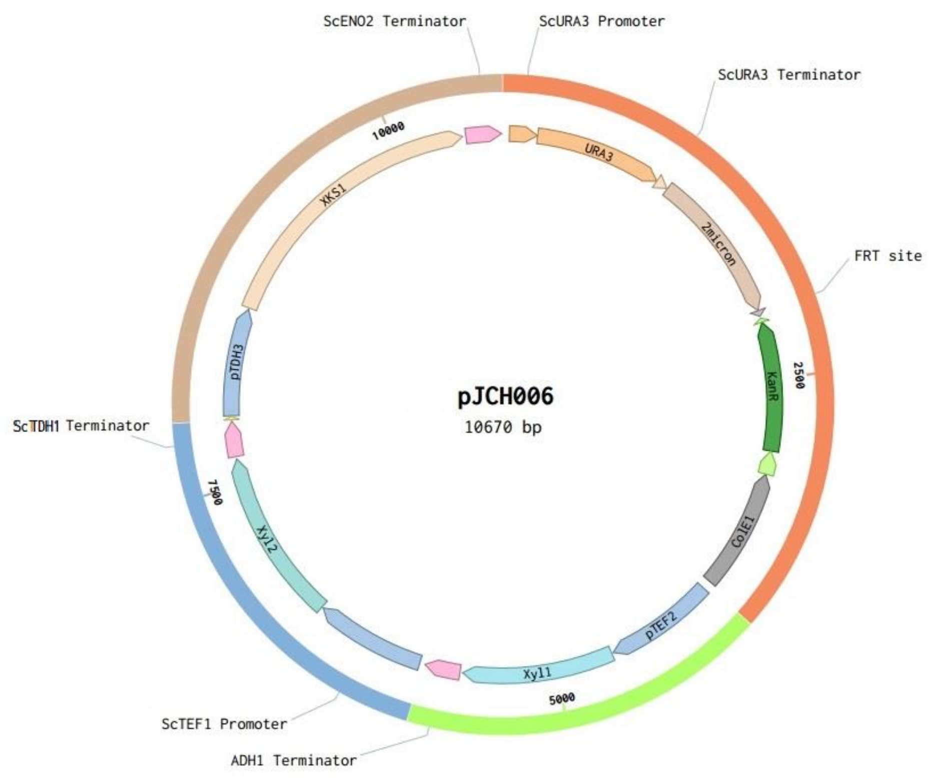

Supplementary Figure 8: Map of plasmid pJCH006. The plasmid map was generated using Benchling (benchling.com). Inner boxes represent plasmid feature and outer boxes represent sub-assembly pieces that were used to construct the plasmid.

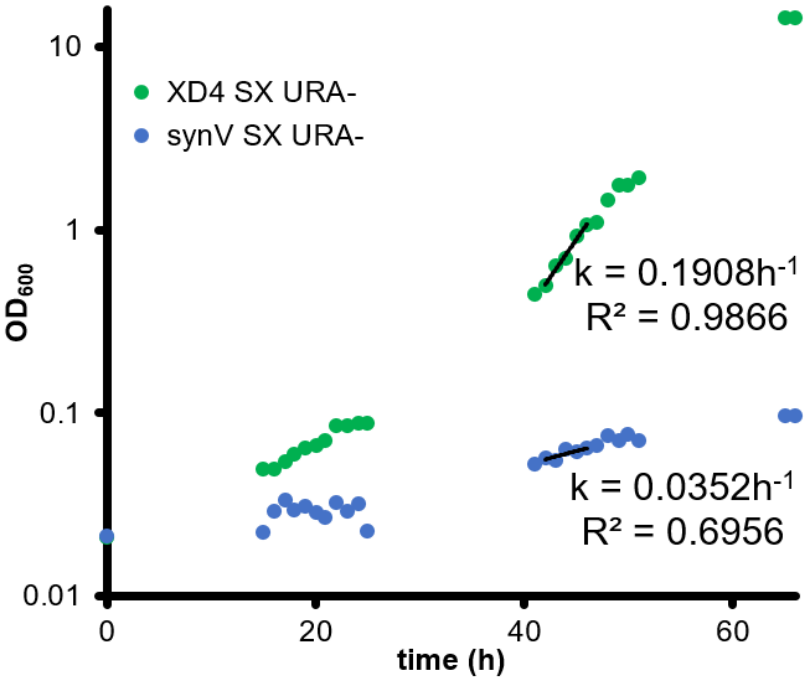

Supplementary Figure 9: Growth of SynV-pJCH006 (blue) and XD4-pJCH006 (green) in SCX URA$^-$ medium in baffled conical flasks. Growth rates (k) were calculated from the curves of best fit shown.

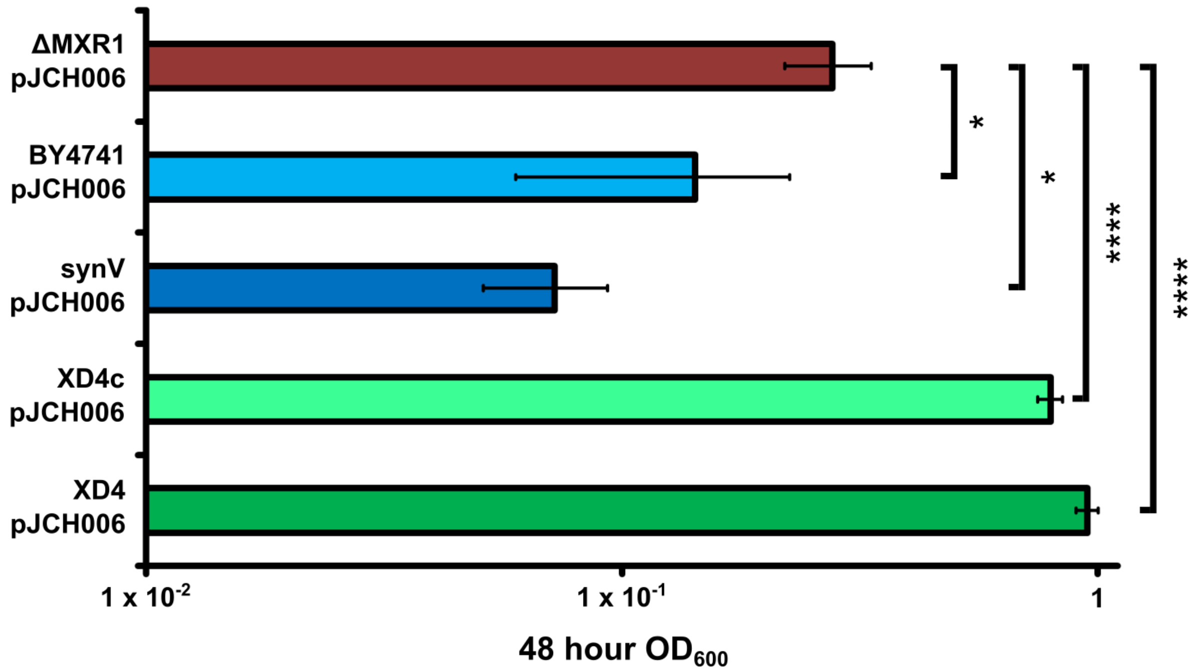

Supplementary Figure 10: Endpoint OD_{600} of XD4 and related strains. Strains were grown in SX URA- in 96-well format for 48 hours and endpoint OD_{600} was taken. Values shown are mean averages of 2 technical replicates of at least 4 biological replicates. Error bars show standard deviation. XD4c-pJCH006 is XD4 cured of pJCH006 and retransformed from the original plasmid stock and ΔMXR1 is a BY4741 strain in which the MXR1 deletion of XD4 has replaced the native MXR1 locus via CRISPR-mediated recombination. Asterisks denote two-tail p value as determined by two-sample t-test, with * denoting p≤0.05, ** denoting p≤0.01, *** denoting p≤0.001 and **** denoting p≤0.0001.

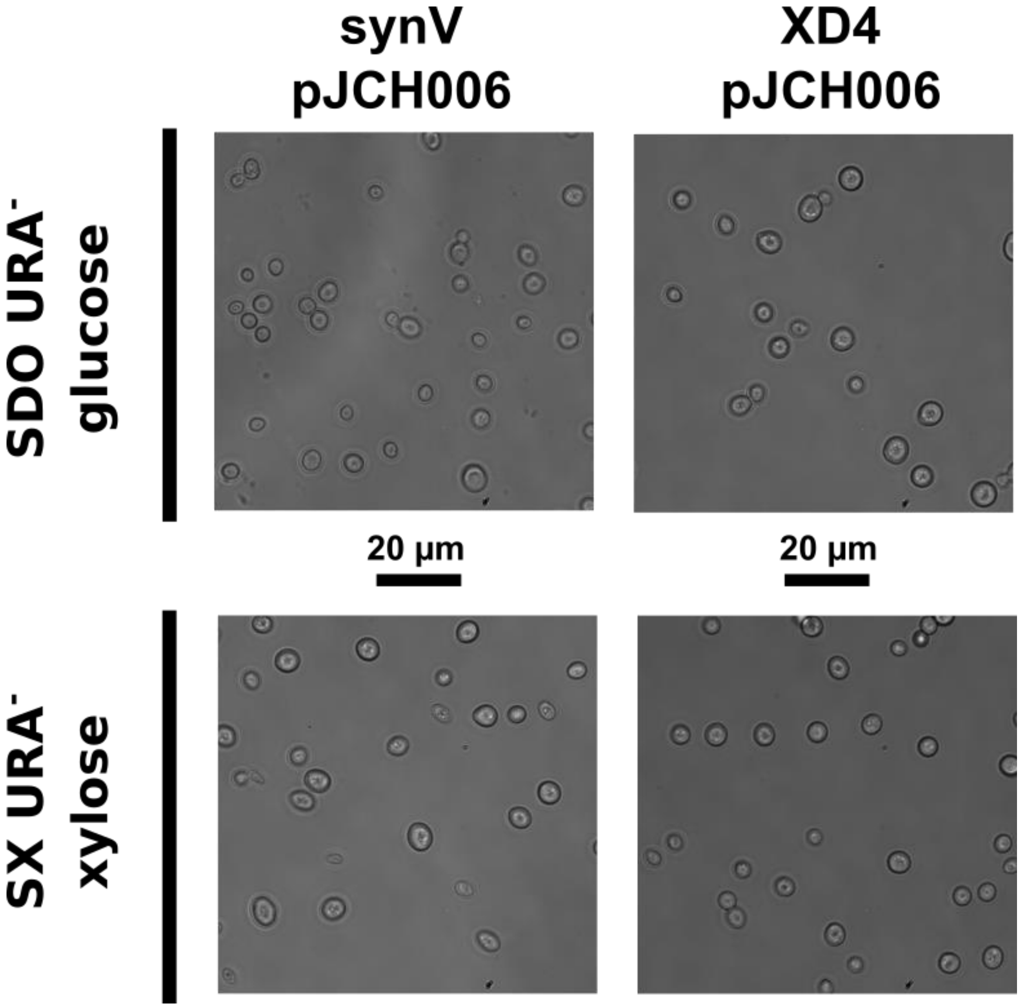

Supplementary Figure 11: Microscopy analysis of strain XD4. Representative microscopy images of synV-pJCH006 and XD4-pJCH006 cultures grown to stationary phase and visualised at 90x magnification.

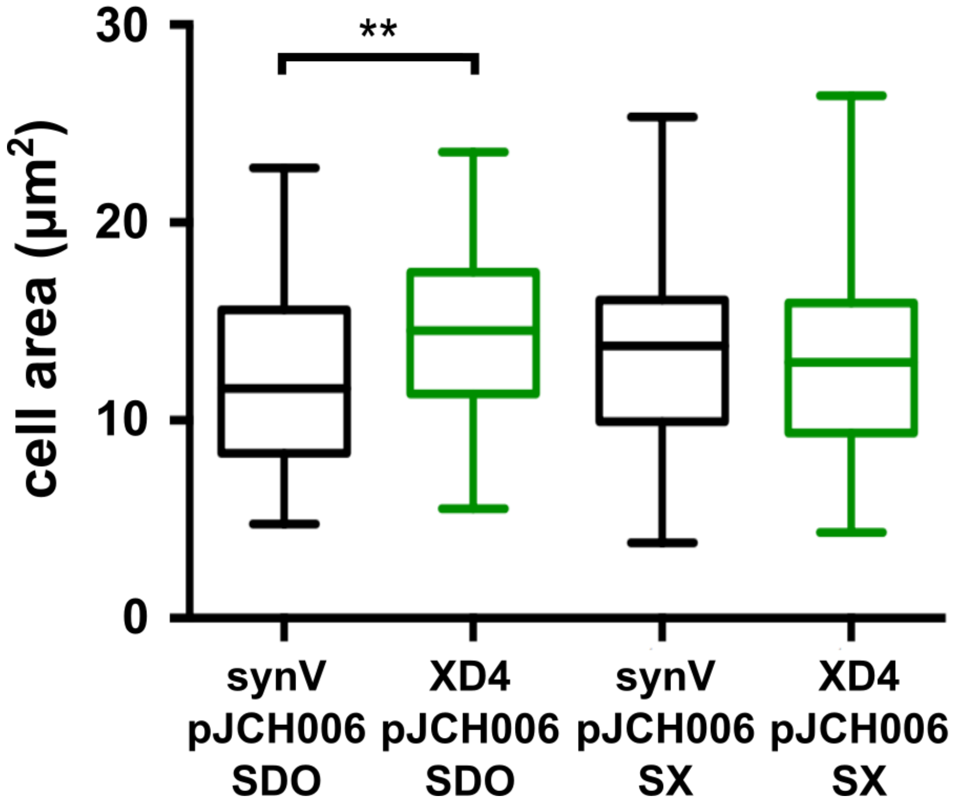

Supplementary Figure 12: XD4 cell area analysis. XD4 and synV cells visualised as in Figure S11 were assessed for cell area. SDO denotes that the cells were grown in SDO URA$^-$ medium and SX denotes that the cells were grown in SX URA$^-$ medium. Top and bottom box edges show the 75th and 25th percentiles with the line between showing the mean average. Bars show the minimum and maximum range of the data points, n≥24 cells. Asterisks denote two-tail p value as determined by two-sample t-test, with * denoting p≤0.05, ** denoting p≤0.01, *** denoting p≤0.001 and **** denoting p≤0.0001.

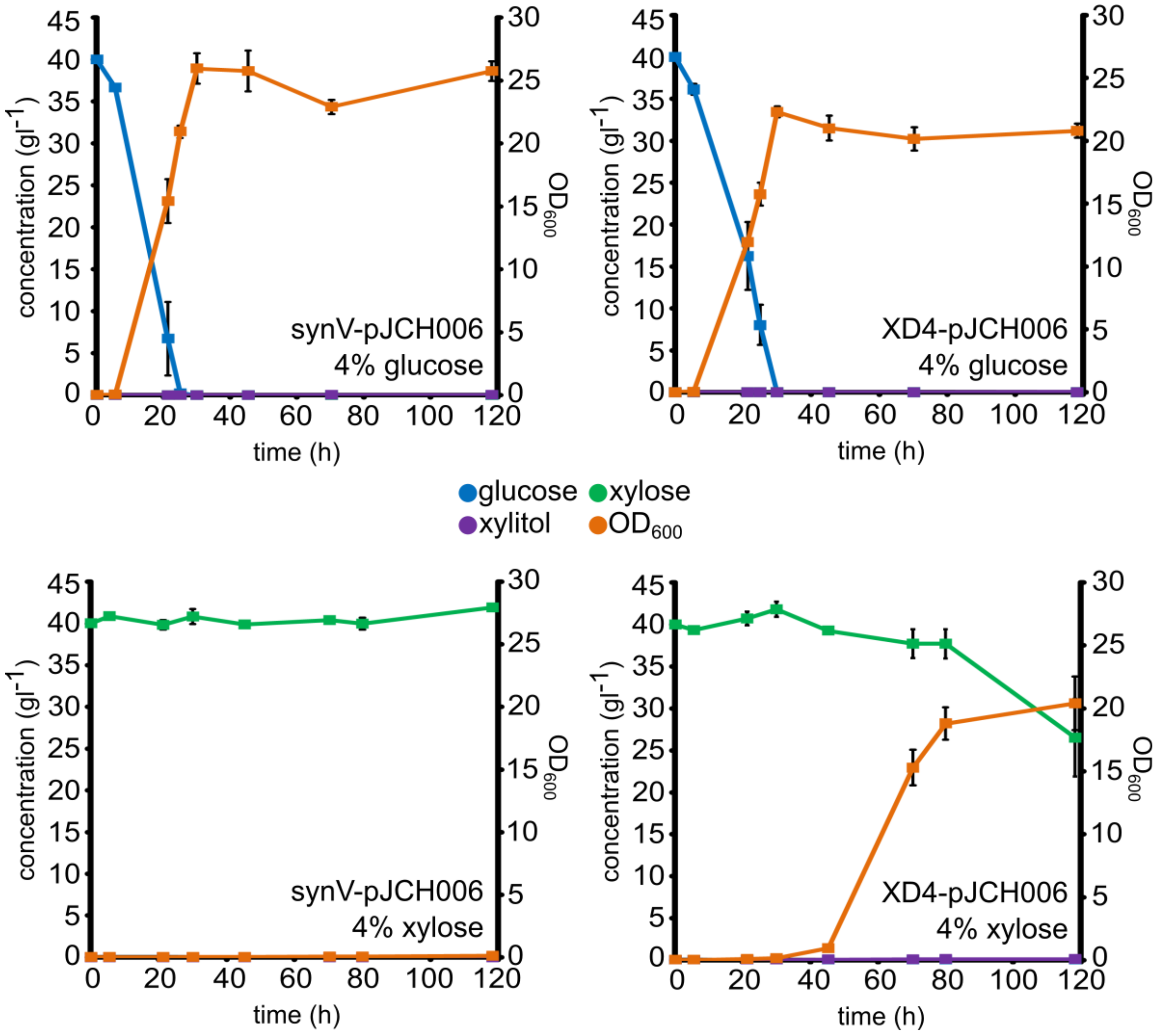

Supplementary Figure 13: HPLC analysis of XD4 and synV cultures. Concentrations of glucose, xylose and xylitol, determined by HPLC, and OD_{600} of synV-pJCH006 and XD4-pJCH006 cultures in SC URA^- medium with either 4% glucose or 4% xylose carbon source. Data plotted are mean averages of 2 biological replicates, error bars denote standard deviation from the mean.

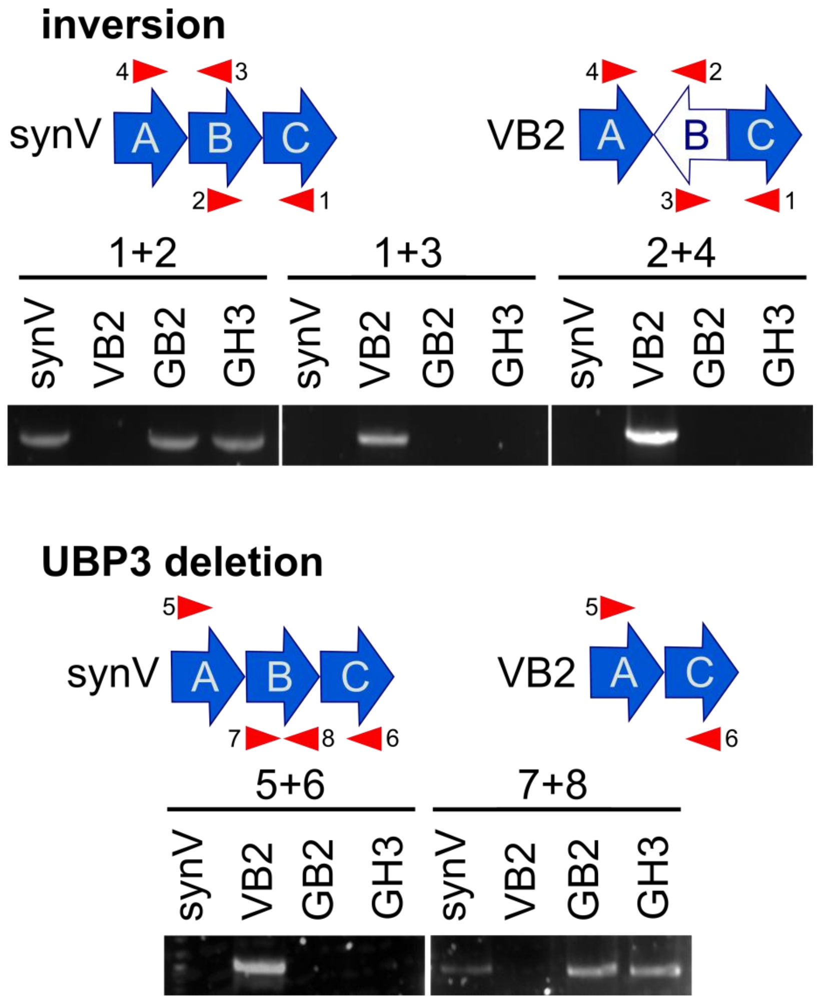

Supplementary Figure 14: PCR confirmation of VB2 SCRaMbLE events identified by nanopore sequencing analysis. "inversion" shows PCR verification of the 5.3 kb inversion in the VB2 strain, "UBP1 deletion" shows PCR verification of the UBP1 deletion in the VB2 strain. Arrows represent sequences between loxPsym sites, numbered triangles represent primer target sites where 1 is GG054, 2 is GG052, 3 is GG075 and 4 is GG051, 5 is GG047, 6 is GG048, 7 is GG049 and 8 is GG050. PCR products were separated by agarose gel electrophoresis and visualised under UV light.

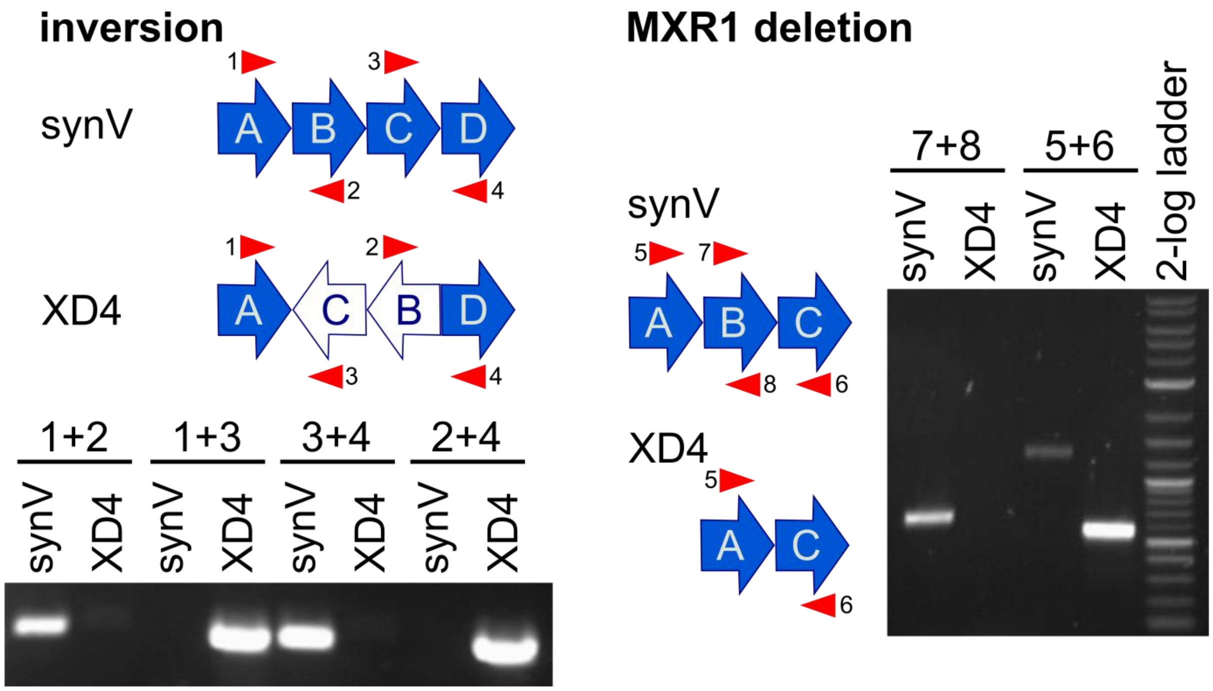

Supplementary Figure 15: PCR confirmation of XD4 SCRaMbLE events identified by nanopore sequencing analysis. "inversion" shows PCR verification of the 7kb inversion in the XD4 strain, "MXR1 deletion" shows PCR verification of the MXR1 deletion in the XD4 strain. Arrows represent sequences between loxPsym sites, numbered triangles represent primer target sites where 1 is GG031, 2 is GG032, 3 is GG033 and 4 is GG034, 5 is GG035, 6 is GG036, 7 is GG037 and 8 is GG038. PCR products were separated by agarose gel electrophoresis and visualised under UV light. "2-log ladder" is New England Biolabs 2-Log DNA Ladder (0.1-10.0 kb).

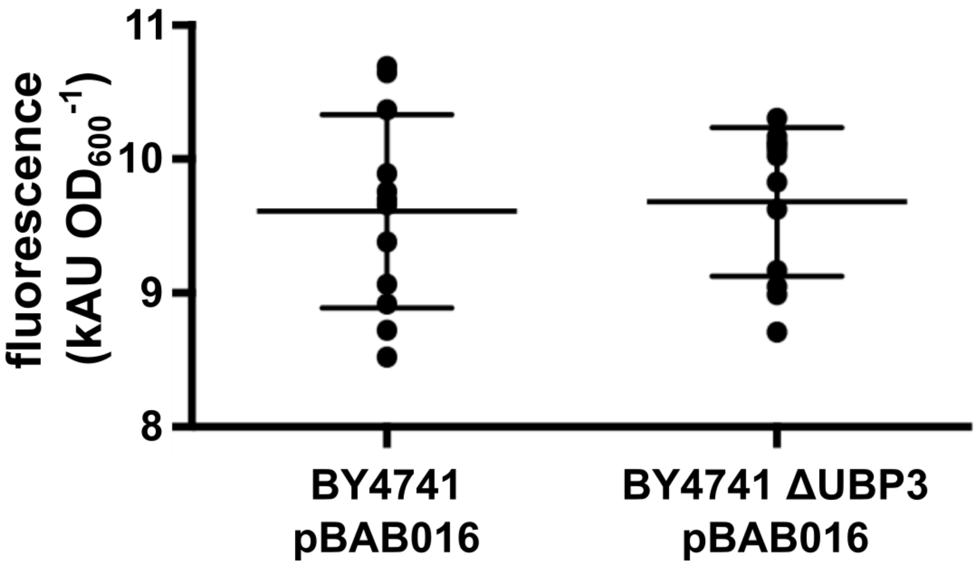

Supplementary Figure 16: sfGFP output of BY4741 ΔUBP3-pBAB012. Comparison of endpoint sfGFP fluorescence output of BY4741-pBAB012 and BY4741 ΔUBP3-pBAB012 after 24 hours growth in 96-well format at 30 °C. Data points represent fluorescence at 520 nm divided by the OD_{600} of the culture at that time point. All OD_{600} values were corrected for medium OD_{600} and all fluorescence values were corrected for medium fluorescence. Horizontal lines show the mean average with error bars representing 1 standard deviation from the mean, n=12 biological replicates.

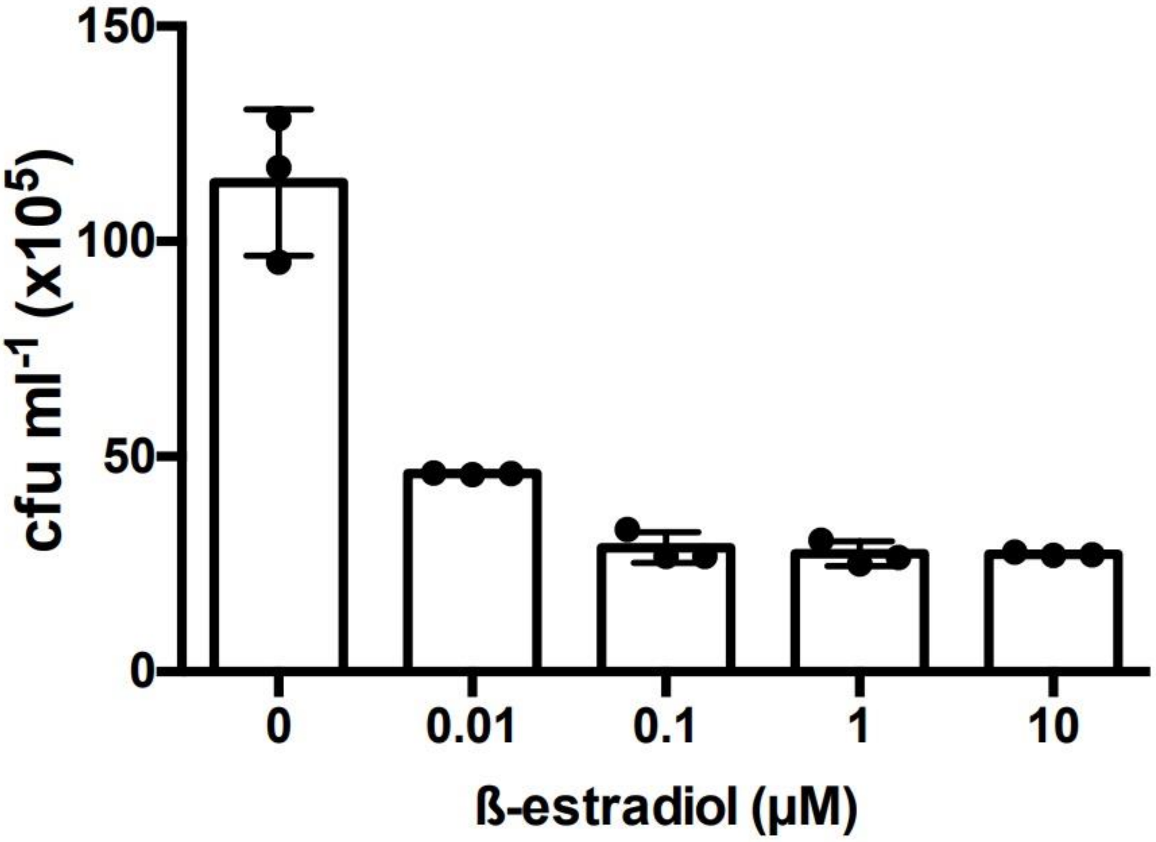

Supplementary Figure 17: Loss in synV-pJCH006 viability caused by varying concentrations of inducer. SCRaMbLE was induced by addition of 0, 0.01, 0.1, 1 or 10 µM β-estradiol for 4 hours, after which cells were washed, serially diluted and plated onto non-selective YPD agar medium. Bars show mean values, error bars denote standard deviation, n=3.

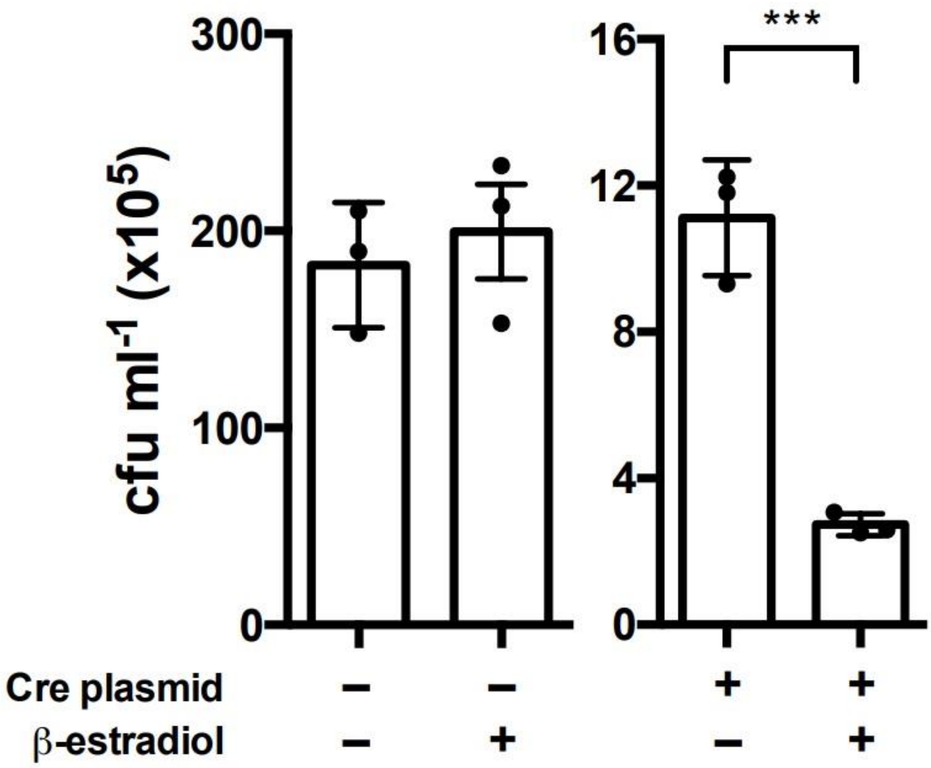

Supplementary Figure 18: Loss in synV-pJCH006 viability caused by 4 hours of SCRaMbLE. SCRaMbLE was induced by addition of 1 μM β-estradiol for 4 hours, after which cells were washed, serially diluted and plated onto non-selective YPD agar medium. Mean values are plotted, error bars denote standard deviation, n=3. Asterisks denote two-tail p value as determined by two-sample t-test, with * denoting $p \leq 0.05$, ** denoting $p \leq 0.01$, *** denoting $p \leq 0.001$ and **** denoting $p \leq 0.0001$.
