# Supplementary material for: Rapid host strain improvement by in vivo rearrangement of a synthetic yeast chromosome
Source: Nat Commun. 2018 May 22;9:1932. doi: 10.1038/s41467-018-03143-w (PMC5964169; doi:10.1038/s41467-018-03143-w)
Supplement: Supplementary file 3 — Description of Additional Supplementary Files [file 41467_2018_3143_MOESM3_ESM.pdf]

Description of Additional Supplementary Files

File Name: Supplementary Data 1
Description: This file contains annotated Genbank files for all plasmids generated in this study
